# High-Temperature Tensile Behaviour of GTAW Joints of P92 Steel and Alloy 617 for Two Different Fillers

**DOI:** 10.3390/ma16175880

**Published:** 2023-08-28

**Authors:** Amit Kumar, Sachin Sirohi, Shailesh Mani Pandey, Pradeep Kumar, Dariusz Fydrych, Chandan Pandey

**Affiliations:** 1 Mechanical Department, Indian Institute of Technology Jodhpur, N.H. 62 Nagaur Road, Karwar 342037, India; kumar.175@iitj.ac.in (A.K.); jscpandey@iitj.ac.in (C.P.); 2 Mechanical Department, SRM Institute of Science and Technology, Delhi NCR Campus, Modinagar 201204, India; 3 Department of Mechanical Engineering, National Institute of Technology, Patna 800005, India; 4 Mechanical and Industrial Engineering Department, Indian Institute of Technology Roorkee, Roorkee 247667, India; 5 Institute of Manufacturing and Materials Technology, Faculty of Mechanical Engineering and Ship Technology, Gdańsk University of Technology, Gabriela Narutowicza Street 11/12, 80-233 Gdańsk, Poland

**Keywords:** dissimilar weld, ER62S-B9, ERNiCrCoMo-1, alloy 617, P92 steel, fracture surface, high-temperature

## Abstract

This study explores the high-temperature (HT) tensile rupture characteristics of a dissimilar gas-tungsten-arc-welded (GTAW) joint between P92 steel and Alloy 617, fabricated using ER62S-B9 and ERNiCrCoMo-1 fillers. The high-temperature tensile tests were performed at elevated temperatures of 550 °C and 650 °C. An optical microscope (OM) and a field emission scanning electron microscope (FESEM) were utilized to characterize the joint. The high-temperature test results indicated that the specimen failed at the P92 base metal/intercritical heat-affected zone (ICHAZ) rather than the weld metal for the ERNiCrCoMo-1(IN617) filler. This finding confirmed the suitability of the joint for use in the Indian advanced ultra-supercritical (A-USC) program. The fracture surface morphology and presence of precipitates were analysed using an SEM equipped with energy dispersive spectroscopy (EDS). The appearance of the dimples and voids confirmed that both welded fillers underwent ductile–dominant fracture. EDS analysis revealed the presence of Cr-rich M_23_C_6_ phases, which was confirmed on the fracture surface of the ER62S-B9 weld (P92-weld). The hardness plot was analysed both in the as-welded condition and after the fracture.

## 1. Introduction

Advanced ultra-supercritical (A-USC) power plants can significantly enhance efficiency, reduce fuel consumption, and lower harmful gas emissions. A-USC plants are being promoted for energy generation in Europe, the US, Japan, China, and India. Ni-based alloys are utilized in A-USC plants for steam temperatures above 700 °C, while martensitic steels (9–12% Cr) are used for lower temperature zones up to 620 °C and 650 °C, respectively [1]. Ni-based alloys, including Alloy 617, Alloy 625, Alloy 740, and Alloy 263, are commonly utilized in turbines, pipes, headers, and tubes for high-temperature components [2,3,4]. Alloy 617 is considered an ideal material for temperatures above 700 °C due to its exceptional resistance to oxidation and corrosion. However, due to the higher cost of the Ni-based alloys, inexpensive ferritic-grade steel such as P91/P92 BM is preferred for components operating at temperatures below 620 °C [5].

The major problem associated with the dissimilar metal joining (DMJ) of Alloy 617 and P92 steel is a mismatch in the coefficient of thermal expansion (CTE), chemical composition, melting point, and microstructure. The mismatch in CTE leads to residual stress at the interface during welding. The linear CTE at 700 °C is calculated as 13.1 µ/K for P92 [6], and 14.8 µ/K for Alloy 617 at the same temperature [7]. The CTE of Alloy 617 is approximately 11.5% more than the CTE of P92 steel. The other major issue faced during the DMJ is the filler wire selection and carbon migration across the interface. The carbon migration produces a carbon-depleted zone which reduces the mechanical properties of the joint at high-temperature applications [8,9]. The carbon migration can be reduced to some extent by using a Ni-based filler with or without buttering in dissimilar metal joining. Lee et al. [10] also use the buttering layer concept of Thyssen-617 filler on the P92 side to join P92 steel and the Inconel 617 alloy to reduce migration. The long-term creep failure at the outer edge of the HAZ (FGHAZ nearer to ICHAZ, or ICHAZ) of the dissimilar weld joint manifests as a type IV failure [11]. A type IV creep failure mostly occurs at high temperatures and lower stress levels in dissimilar welded joints. The heterogeneity in the microstructure and the very first residual stress in the weldment are the main causes of type IV failure [12]. Another reason for the formation of type IV cracks is the Laves phase formation (rich in Mo and W) at grain boundaries under high-temperature, low-stress conditions for long-term applications. Wang et al. [13] also confirmed heterogeneity in the microstructure at the FGHAZ near the ICHAZ, and the formation of the coarse Laves phase is the main reason for type IV failures in P92 steel weldments. High-temperature (HT) and room temperature (RT) tensile behaviour were also investigated by several authors to understand the issues related to DMJs. Sah et al. [14] analysed the HT tensile test along with creep rupture tests on similar weld samples of diffusion-welded Alloy 617 plates at seven different temperatures ranging from 100 °C to 950 °C. Zang et al. [15] investigated the tensile properties of 9CrMo steel at room temperature, 400 °C, and 550 °C to better understand grain elongation during tensile tests and optimize the composition of 9CrMo steel. Wang et al. [16] observed serrated behaviour in the graph during HT tensile tests at 640 °C for the filler ERNiCr-3 GTAW joint of P92 and HR3C. The recrystallization and precipitation of Cr_23_C_6_ carbides were likely responsible for the serrated behaviour. Chen et al. [17] investigated RT and HT (575–650 °C) tensile experiments on dissimilar welds of P92 and TP347H steel with filler ERNiCr-3 as the weld metal. Failure of the HT samples at FGHAZ was attributed to the fact that TP347H exhibits a dispersion-strengthening effect at elevated temperatures. Zang et al. [18] evaluated RT and HT (650 °C) tensile properties and the microstructure of the GTAW-welded joint of austenitic 22Cr15Ni3.5Cu steel with filler ERNiCrCoMo-1 weld metal. During both tests, the interfacial fractured joints experienced ductile failure with slipping separation. Further post-tensile test microstructure studies of the welded joint revealed the presence of precipitates of different sizes. The authors also noted that 10^−9^ scale precipitates assist in providing HT resistance to the welded joint. In addition to RT and HT tensile behaviour, most of the authors focused on the creep rupture behaviour of weld joints involving 9%Cr and austenitic steel or Inconel alloy [19,20,21].

From the literature review, it has been found that high-temperature tensile behaviour is an important study for evaluating the performance of dissimilar weld joints (DWJ). There is a lack of research on the high-temperature behaviour of DWJ between the Inconel 617 alloy and P92 steel using ER62S-B9 and ERNiCrCoMo-1 as filler material. Thus, a study was conducted to investigate the microstructure, microhardness, and tensile properties of the weldment at temperatures commonly found in A-USC power plant components, specifically at 550 °C and 650 °C.

## 2. Experimental Details

**Figure 1:**
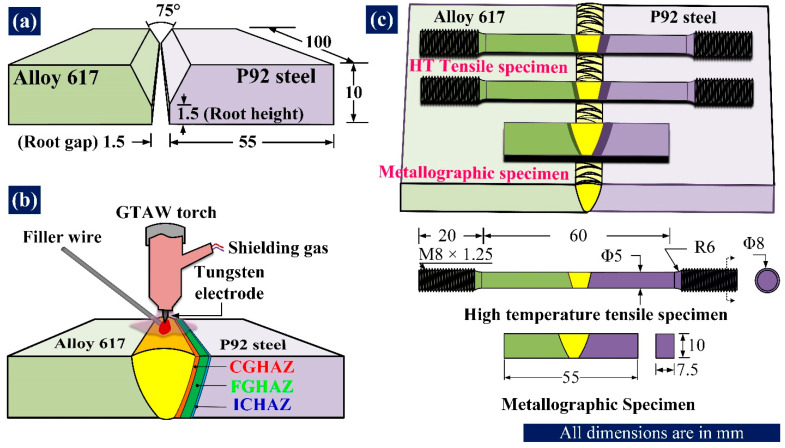
Schematic of (**a**) V-groove, (**b**) GTAW joint, (**c**) Welded plate and extracted samples with dimensions.

For this study, two 10 mm thick plates of P92 steel and Alloy 617 were welded together using the multi-pass gas tungsten arc welding (GTAW) process. The plates were cut and machined into dimensions of 100 mm × 55 mm × 10 mm. One side of the plates was machined as per the conventional V-groove of bevel angle 37.5° with a root face of 1.5 mm. All required dimensions related to the groove are illustrated in Figure 1a. Two joints were made: one joint with the P92 filler (ER62S-B9), and the other joint with the ERNiCrCoMo-1 filler. The chemical compositions of plates and fillers are mentioned in previously published articles [22,23]. Each joint was completed in five passes, including the root pass. The root pass was completed with a welding current of 110 A and voltage of 12 V with a travel speed of 80 mm/min. For the remaining four passes, the welding current and voltage were 120–125 A and 12–14 V, respectively, with a travel speed of approximately 50–60 mm/min [22,23]. The argon gas was used for shielding purposes with a flow rate of 15 L/min. The P92 side HAZs include the region of the coarse grain heat-affected zone (CGHAZ), fine grain heat-affected zone (FGHAZ), and intercritical heat-affected zone (ICHAZ), all of which are shown in Figure 1b. To study the metallurgical and mechanical properties of the weldments, transverse samples were extracted from the plates (Figure 1c) using wire-cut electrical discharge machining (EDM). The metallurgical samples underwent grinding and polishing with sandpaper of grit sizes varying from 180 to 2000. To achieve a mirror surface finish, the sandpaper-grinded samples were further polished with alumina powder. Polished samples were then etched with Villella’s reagent for 60 s to reveal the microstructure of the P92 steel BM and P92 weld. The electrolytic etching was performed in a 10% oxalic acid solution to reveal the microstructure of Alloy 617 BM and the IN617-weld. The etched samples were analysed with an optical light microscope (model DMC4500 Leica) and scanning electron microscopes (model Apreo-2S Thermoscientific). The dimensions of the HT tensile test specimen and metallurgical specimen are displayed in Figure 1c. The HT tensile tests were performed at 550 °C and 650 °C, respectively, at a constant cross-head speed of 1 mm/min. The tensile tests were carried out on the universal testing machine ‘Shimadzu AG-X-100KN’. The microhardness of the HT tensile samples was determined on a Vickers hardness tester (model AVHD-1000XY Banbros) along with the sample’s longitudinal axis by applying a load of 500 g for a duration of 10 s. The indent was taken at every 0.5 mm gap. For the sake of convenience in this study, the P92-weld samples tested at 550 °C and 650 °C are represented as P92-weld-550 °C and P92-weld-650 °C, respectively. The IN617-weld samples tested at 550 °C and 650 °C are represented as IN617-weld-550 °C and IN617-weld-650 °C, respectively.

## 3. Result and Discussion

### 3.1. Characterization of Base Metals and HAZ and Interface

**Figure 2:**
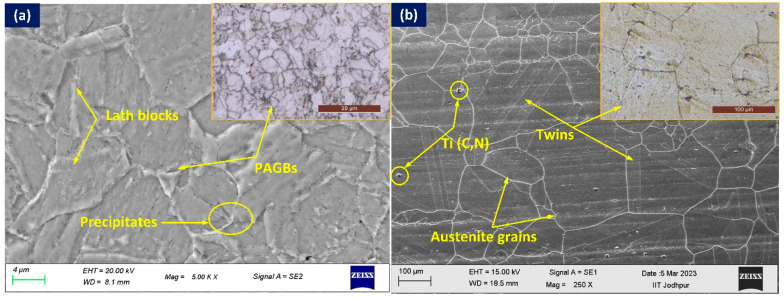
SEM and optical microstructure of (**a**) P92 steel BM and (**b**) Alloy 617 BM.

The microstructure of P92 steel BM is depicted in Figure 2a. The tempered martensitic microstructure consists of lath blocks, lath packets, packet boundaries, and prior austenite grain boundaries (PAGBs). The microstructure is strengthened through the presence of Cr, Mo, and W-rich coarse M_23_C_6_ precipitates, as well as V and Nb-rich fine MX (X: C, N) precipitates [24,25]. The microstructure of Alloy 617 BM consists of a solid solution that strengthens the matrix of austenite grain (Figure 2b) along with twins and carbides of Cr and Mo (M_23_C_6_), Mo-rich (M_6_C), and Ti-rich (MX (X: C, N)) [24,25].

**Figure 3:**
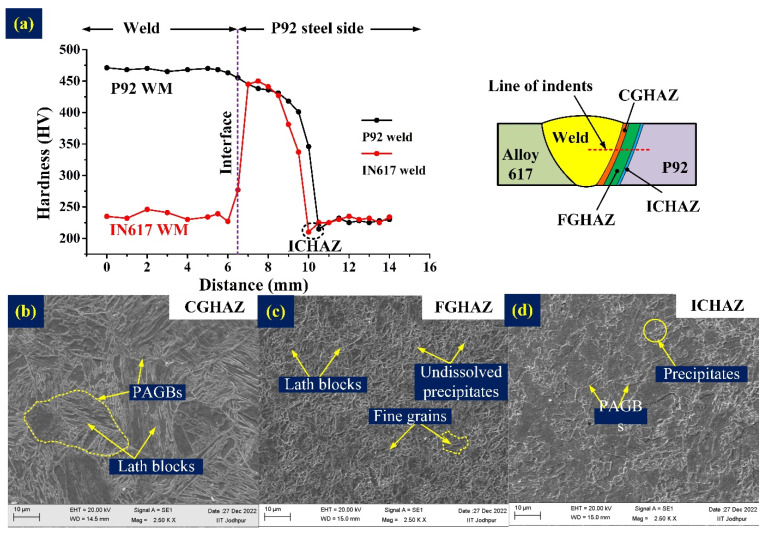
(**a**) Vickers-microhardness profile across the welded joint and SEM image of (**b**) CGHAZ, (**c**) FGHAZ, and (**d**) ICHAZ.

The region of P92 HAZ near the interface, i.e., CGHAZ, experiences a temperature far above the upper critical temperature (Ac_3_), characterized by coarse PAGs, as depicted in Figure 3b. The high temperature causes carbide precipitates to dissolve, resulting in the formation of coarse PAGs and an untempered martensitic matrix with higher levels of carbon and nitrogen. This is also reflected in the hardness plot mentioned in Figure 3a. In contrast, the FGHAZ adjacent to the CGHAZ experiences peak temperatures close to or higher than Ac_3_, causing incomplete dissolution of carbide precipitates and resulting in fine PAGs, also shown in Figure 3c. The hardness of the FGHAZ measured lower than that of the CGHAZ, as shown in the hardness plot in Figure 3a. ICHAZ is formed near the base metal and experiences a temperature between the lower critical temperature (A_c1_) and Ac_3_ that results in a negligible dissolution of the precipitates. The over-tempering of ICHAZ results in coarsening of the precipitates that reduces the strength and hardness of the region, and it is marked as the region of lowest hardness, as mentioned in Figure 3a; the microstructure of ICHAZ is shown in Figure 3d. The area located beyond the ICHAZ from the fusion line does not undergo a transformation from austenite to martensite during welding. The microhardness profiles for the P92-weld and IN617-weld samples are shown in Figure 3a. In the P92-weld sample, the average hardness of WM was 468 ± 3 HV, while in the IN617-weld sample, the average hardness of WM was 236 ± 6 HV, which is approximately half of the hardness of the P92-weld. In the P92-weld sample, the hardness of CGHAZ, FGHAZ, and ICHAZ was 442 ± 5 HV, 406 ± 36 HV, and 218 HV, respectively [22]. In the IN617-weld sample, the hardness of CGHAZ, FGHAZ, and ICHAZ was 448 ± 4 HV, 397 ± 47 HV, and 214 HV, respectively [23].

**Figure 4:**
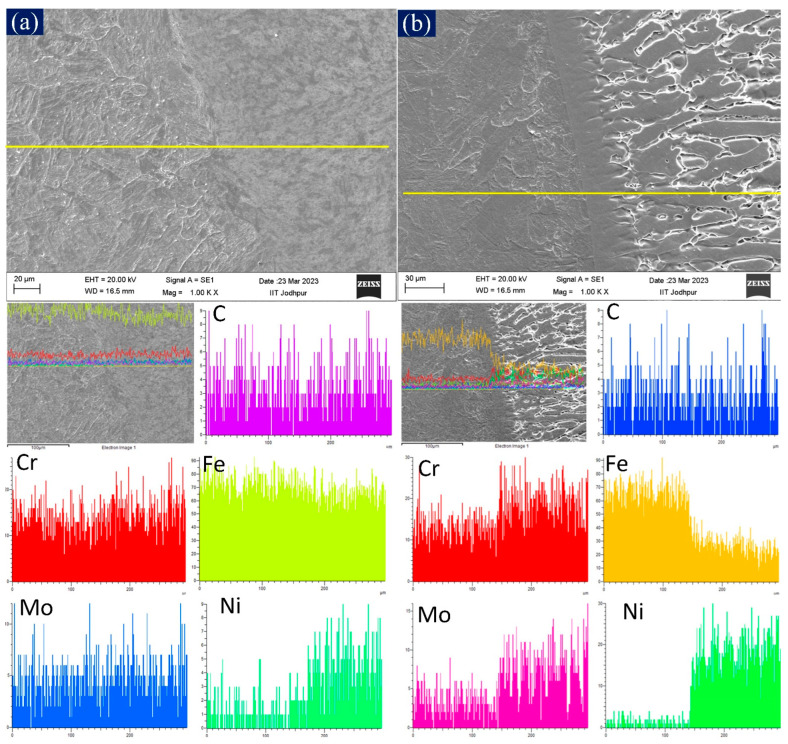
EDS line map across the interface of (**a**) P92-weld and P92 BM (**b**) IN617-weld and P92 BM.

The EDS line map was carried out on as-welded samples across the interface of WM and P92 to determine the diffusion of elements from the WM to P92-steel (Figure 4a,b). No significant diffusion was observed for the P92 filler because it has a similar composition to that of the P92 base material (Figure 4a). This confirms that the hardness variation was due to the change in the microstructure of the HAZ of P92, not from the elemental alteration due to diffusion of the elements from the WM to P92-HAZ. However, a significant element variation is seen at the interface of the weld and P92 BM for the ERNiCrCoMo-1 filler (Figure 4b). The major variation is seen for Cr, Mo, Fe, and Ni. The line map confirms the diffusion of Fe from the P92 base matrix to the weld, as well as the diffusion of Cr, Ni, and Mo from the weld to the P92 base matrix. A similar observation has also been made in our previous study from the P92 filler [22] and the IN617 filler [23] welded joint.

### 3.2. HT Tensile Behaviour

**Figure 5:**
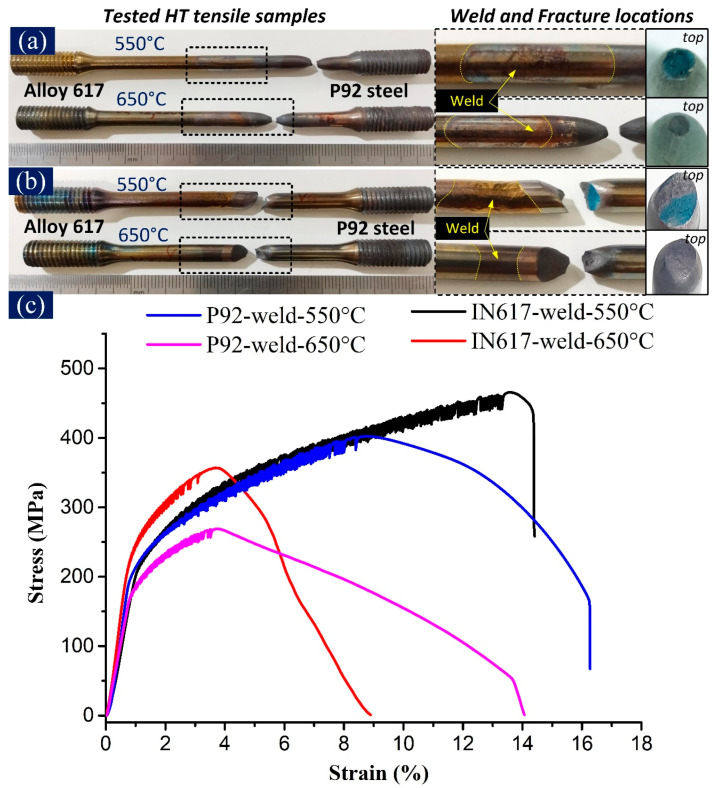
Tested HT tensile samples of (**a**) P92-weld (**b**) IN617-weld, and (**c**) HT tensile stress–strain curves.

The ultimate tensile strength (UTS) of P92 BM was 476 MPa at 550 °C and 319 at 650 °C, respectively. The UTS of the P92-weld-550 °C sample was 402 MPa, which is higher than the UTS (269 MPa) of P92-weld-650 °C. Similarly, the UTS of IN617-weld-550 °C was 466 MPa, which is higher than the UTS (357 MPa) of IN617-weld-650 °C. In another study, the IN617-weld joint of different diameters, the UTS of the welded joint measured 384 MPa and 263 MPa at 550 °C and 650 °C, respectively [23]. Table 1 indicates the UTS, yield strength, percentage elongation, area reduction, and fracture locations of HT tensile samples of the P92-weld and the IN617-weld. At higher temperatures, the thermal energy caused the atoms to vibrate more, which weakened the metallic bonds and resulted in a decrease in the strength and ductility of the material. In all tested samples, the tensile strength at 550 °C was higher than the tensile strength at 650 °C. Rahman et al. [26] and Rao et al. [27] observed curve-maintaining serrated flow behaviour for the strain rate of 10^−4^ orders tested from 600 °C to 800 °C and 300 °C to 700 °C, respectively. Considering the authors’ findings, the HT tensile test was performed by keeping the strain rate in the order of 10^−4^ (2.7 × 10^−4^/s). When Alloy 617 is tested at a high temperature (>500 °C), the serrated flow is caused by the diffusion of substitutional solute Cr, Co, and Mo atoms into the grains [27], where they interact with dislocations to create strain localization. This strain localization causes the formation of dynamic strain ageing (DSA) serration, which are regions of the material where plastic deformation occurs in a series of rapid bursts. At low temperatures, solute atoms are immobile and do not interact with dislocations. However, at higher temperatures, solute atoms become mobile and can cluster around dislocations, which results in an increase in dislocation mobility. This, in turn, causes the dislocations to move more easily and interact with the solute atoms, creating strain localization. The strain localization results in a sudden increase in stress, followed by a decrease in stress as the material undergoes plastic deformation. This leads to the characteristic serrated appearance of the stress–strain curve during HT tensile testing. The serrated flow was observed in all welded samples with stress–strain curves tested at temperatures of 550 °C and 650 °C. Rodriguez [28] named this type of serration ‘type C’ serration. Sudden and repeated irregular stress drops below the overall level of the flow curve are regarded as type C serration. In the case of HT tensile testing of the P92-weld, the serrated flow (Figure 5c) was governed by the Alloy 617 side metal. On the other hand, for IN617-weld samples, the Alloy 617 side metal and nickel-based (ERNiCrCoMo-1) weld metal were responsible for the serrated flow behaviour (Figure 5c). The tested tensile samples of both welded samples, IN617-weld and P92-weld, are shown in Figure 5a,b along with the marked fracture location, weld location, and a top view of the fracture location.

**Figure 6:**
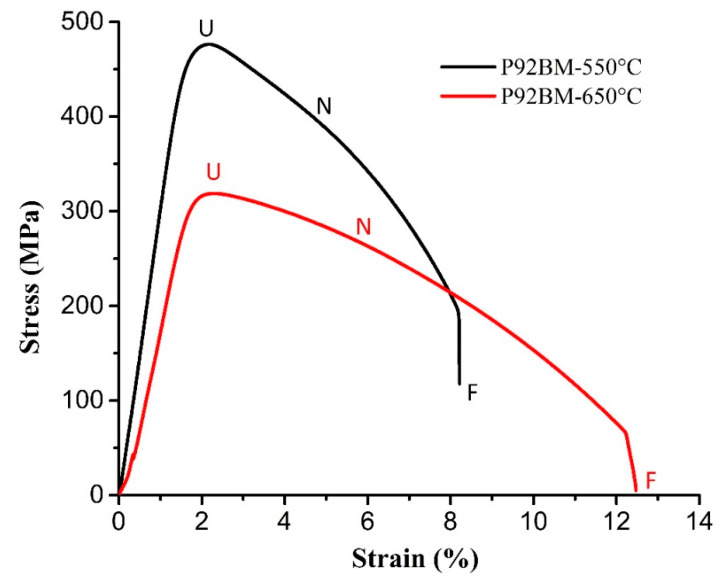
HT tensile characteristic of P92 BM at 550 °C and 650 °C.

**Figure 7:**
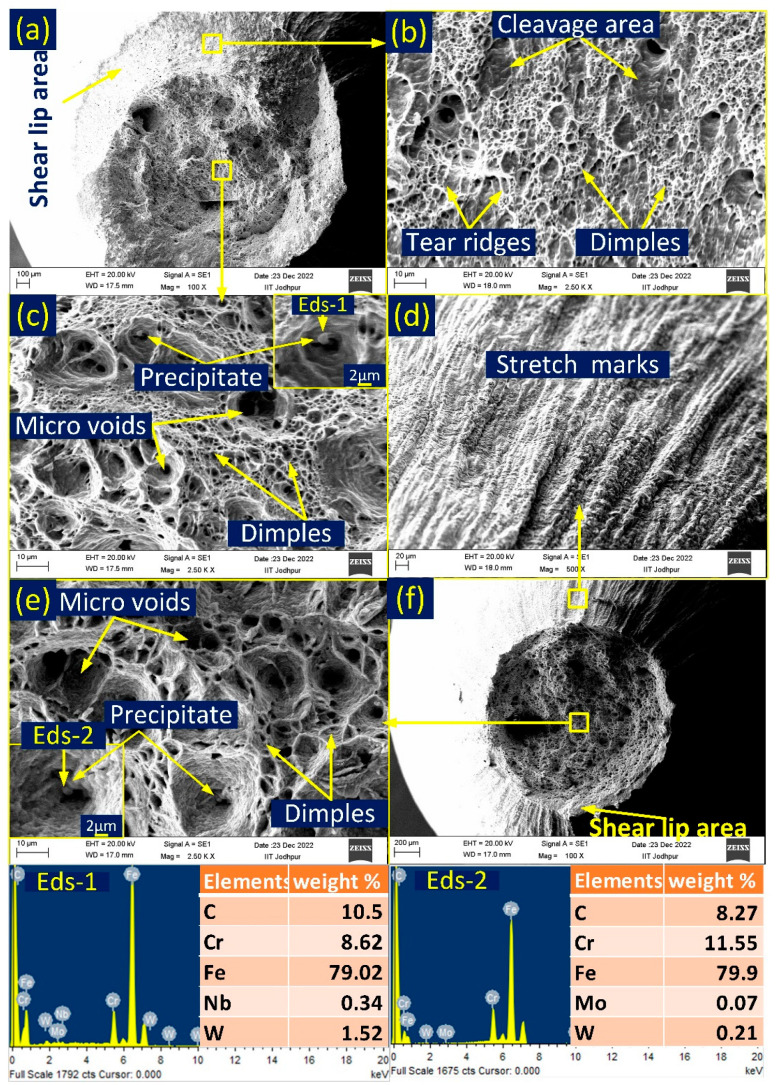
Fractographic SEM images of P92-weld sample (**a**–**c**) tested at 550 °C and (**d**–**f**) tested at 650 °C.

Both P92-weld samples failed at the base metal of P92 steel. Thus, emphasis was also given to the stress–strain characteristic of P92 BM at high temperatures, as shown in Figure 6. During HT tensile testing of P92 BM at temperatures of 550 °C and 650 °C, P92 BM underwent work hardening up to point U (UTS point), followed by prolonged and steady softening from point U to N. During the softening stage, the stress decreased, with this reduction being more pronounced for lower strain rates (10^−4^/s). Once the stress reached point N, a more substantial softening effect occurred, resulting in a change in slope that eventually led to fracture at point F. The material experiences softening at high temperatures, which leads to a decrease in its strength and hardness. This can make the material more susceptible to deformation and failure under stress [22]. The P92-weld sample displayed the same softening behaviour (Figure 5c) as P92 BM (Figure 6), with a softening curve changing the nature at point N, and a slope change up to the fracture point. The shape of the fracture appeared as a cup and cone type of fracture (Figure 7a). The softening stage of IN617-weld-550 °C was the smallest compared to IN617-weld-650 °C. As shown on the graph of IN617-weld-550 °C in Figure 5c, the softening curve (after the UTS point) first displayed ductile behaviour, and then sudden fracture occurred. On the other hand, the IN617-weld-650 °C sample showed smooth and elongated softening behaviour, indicated in Figure 5c. The blue colour in the partial fracture surface is also evidence of the partial brittle fracture in the partial area; an image of the sample is shown in Figure 5b.

The fractography of P92-weld-550 °C and P92-weld-650 °C samples is shown in Figure 7a–c and Figure 7d–f, respectively. At both temperatures, a cup–cone-shaped fracture was observed with shear lips, as shown in Figure 7a,b. The fracture mouth area of P92-weld-650 °C is less than the sample tested at 550 °C. The percentage area reduction is also shown in Table 1. The central region and shear lip of the fracture surface are shown in Figure 7b,c. The central area is mostly filled with dimples combined with microvoids; thus, the material is ductile in nature in the central region. The shear lip area indicates a shallow microvoid, cleavage area, tear ridges, and dimples, which indicates less ductility than the central region. Thus, for P92-weld-550 °C, this indicates a mixed mode of fracture characteristic. On the other hand, the P92-weld-650 °C sample indicates deeper microvoids rather than shallow dimples, and the size of the dimples and microvoids is also larger than the size observed at 550 °C (Figure 7e). From the morphology of the fracture surface, we can infer that a more ductile nature can be seen at 650 °C than the tested 550 °C. When a tensile sample undergoes stress, the stress concentration around the precipitates can cause localized deformation, leading to the formation of microvoids. These microvoids may grow and coalesce, eventually leading to the initiation and propagation of cracks. The point energy dispersive spectroscopy (EDS) was taken at the precipitate observed inside the dimples. Points EDS-1 and EDS-2 confirm that the Cr-rich (M_23_C_6_) carbide secondary phase [25] was observed in the vicinity of the microvoids. The outer surface near the fracture area is also seen via the SEM observation, and the surface was found rough due to axial force; stretch marks are shown in Figure 7d. Stretch marks on the outer surface of the lip can indicate that localized deformation has occurred in this area and that the material has undergone significant elongation or stretching.

**Figure 8:**
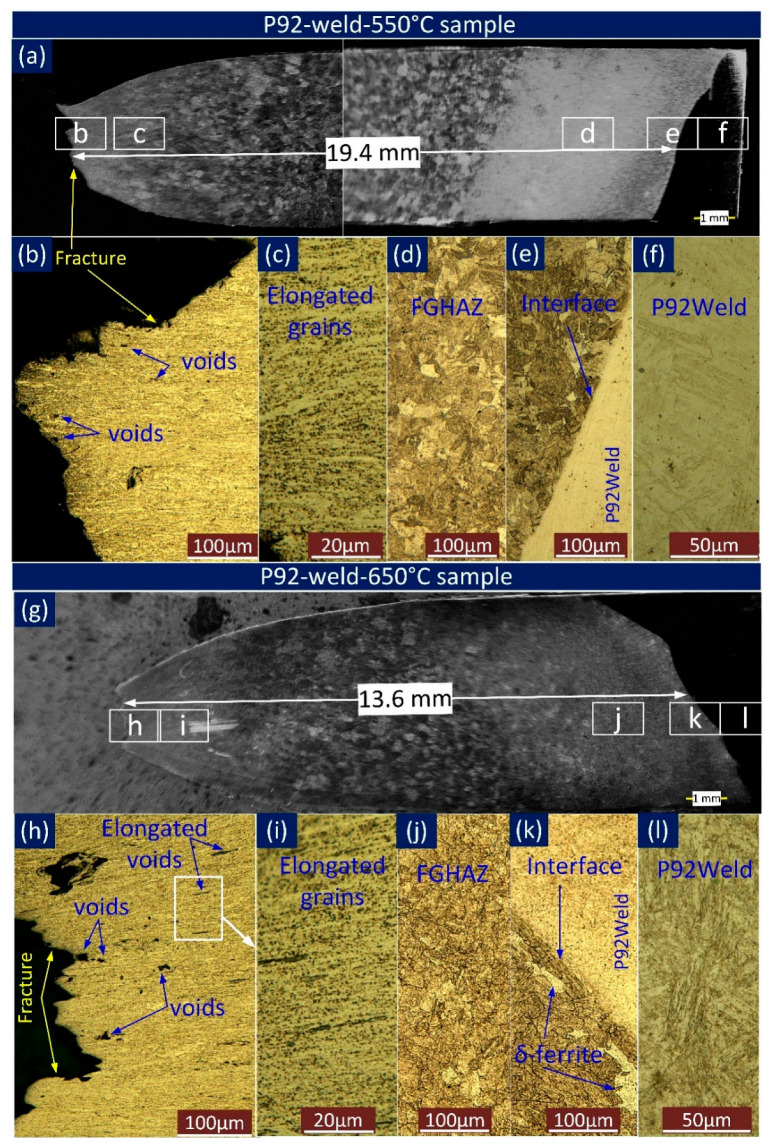
Macrograph and microstructure of longitudinal section of P92-weld sample. (**a**) SEM image of fractured specimen P92-weld-550 °C; optical image of various marked location of (**a**): (**b**) fracture tip(**c**) elongated grains(**d**) FGHAZ (**e**) Interface (**f**) P92 weld (**g**) SEM image of fractured specimen P92-weld-650 °C; optical image of various marked location of (**g**): (**h**) fracture tip (**i**) elongated grains (**j**) FGHAZ (**k**) interface (**l**) P92 weld.

**Figure 9:**
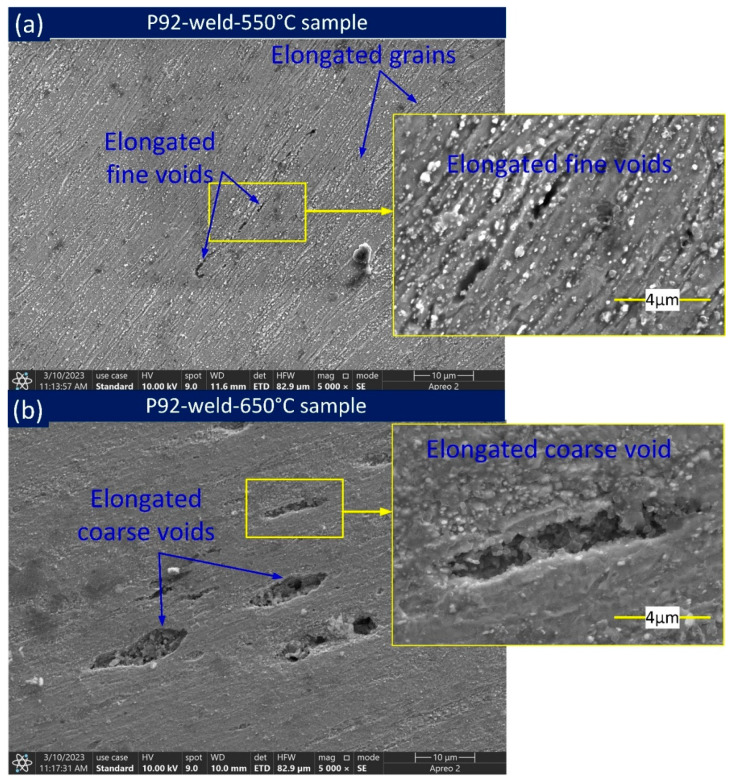
SEM images near fracture location of P92-weld sample tested at (**a**) 550 °C and (**b**) 650 °C.

The macrograph in Figure 8a,g indicates the fracture location is 19.4 mm from the weld interface for P92-weld-550 °C and 13.6 mm for P92-weld-650 °C. Larger-sized voids are seen near the fracture location in P92-weld-650 °C than in P92-weld-550 °C, as depicted in Figure 8c,i. The grains are elongated near the fracture location and are clearly seen in Figure 8b,h. Noticeably elongated voids are seen within the elongated grain in P92-weld-650 °C, whereas no elongated voids are seen in the optical image in P92-weld-550 °C. No appreciable void or gap was noted at the P92-weld interface (Figure 8e,k) in both P92-weld-550 °C and P92-weld-650 °C samples. Furthermore, Figure 8d,j indicates no sign of appreciable voids at the FGHAZ and weld centre region in Figure 8f,l. The near-fracture areas of P92-weld-550 °C and P92-weld-650 °C samples are further characterized. The SEM image (Figure 9a) confirms the elongated fine voids appeared at the near-fracture area of the P92-weld-550 °C sample. The coarse-size elongated void appeared (Figure 9b) at the near-fracture area of the P92-weld-650 °C sample.

**Figure 10:**
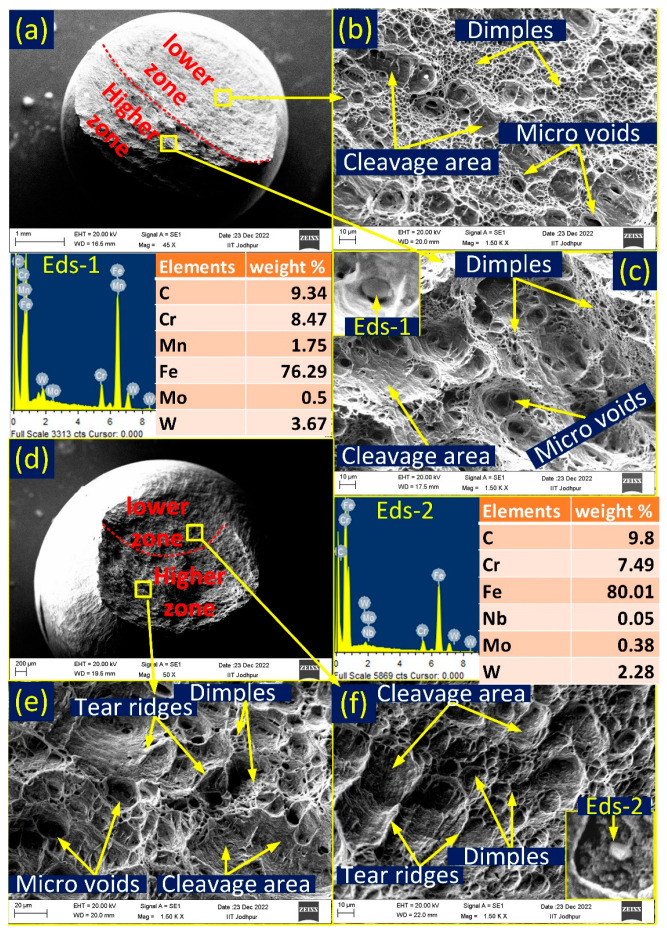
Fractographic SEM images of IN617-weld sample tested at 550 °C (**a**) top view, (**b**) lower zone detailed view, (**c**) higher zone detailed view and EDS of particles; sample tested at 650 °C, (**d**) top view, (**e**) higher zone detailed view, (**f**) lower zone detailed view and EDS of particles.

The fracture morphology of IN617-weld-550 °C and IN617-weld-650 °C samples is shown in Figure 10a–c and Figure 10d–f, respectively. The fracture surface of both samples is divided into two zones: a lower zone and a higher zone. The lower fracture zone of IN617-weld-550 °C indicated a mixed mode (ductile + brittle) of fracture; microvoids, more dimples, and cleavage areas in Figure 10b show evidence of a mixed mode of failure. In the higher fracture zone, (Figure 10c) microvoids, less dimples, and cleavage area indicated a dominated mixed fracture mode. The IN617-weld-650 °C lower fracture zone (Figure 10e) specified shallow dimples and cleavage area, which indicates a brittle fracture mode, but the higher zone showed a mixed mode of fracture with microvoids, deep dimples, and cleavage areas, as evidenced in Figure 10f. From the morphology of the fracture, it can be concluded that the IN617-weld sample shows more ductile behaviour at 550 °C than at 650 °C during a test. It also provided good agreement with the observation made from the stress–strain curve (Figure 5c); the percentage of elongation of IN617-weld-550 °C was higher than the percentage of elongation of IN617-weld-650 °C, as shown in Table 1. The voids are nucleated from the secondary phase’s particles noticed in the fracture SEM images. Point EDS on the particles in the voids confirm the presence of Cr-rich carbide precipitate in the vicinity of voids. EDS-1 and EDS-2 confirm Cr-rich secondary phase presence in the vicinity of large voids seen in fracture images of IN617-weld-550 °C and IN617-weld-650 °C, respectively.

**Figure 11:**
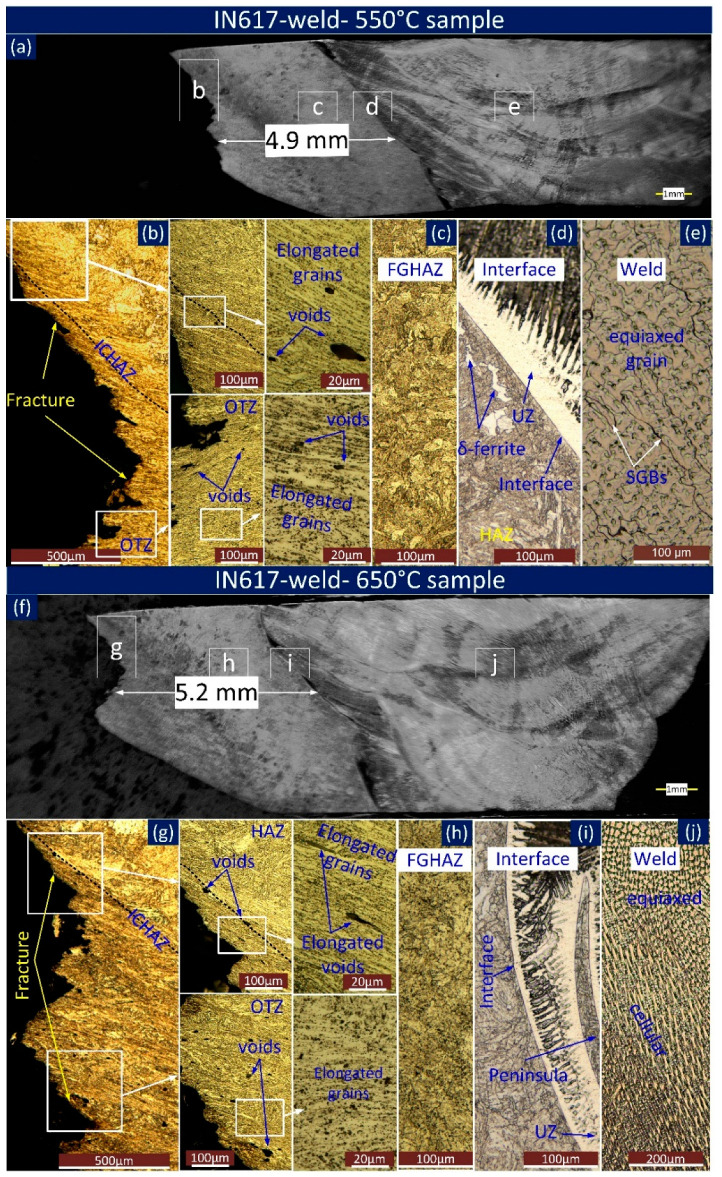
Macrograph and microstructure of longitudinal section of IN617-weld sample. (**a**) SEM image of fractured specimen IN617-weld-550 °C; optical image of various marked location of (**a**): (**b**) fracture tip (**c**) FGHAZ (**d**) Interface (**e**) weld; (**f**) SEM image of fractured specimen IN617-weld-650 °C; optical image of various marked location of (**f**): (**g**) fracture tip (**h**) FGHAZ (**i**) interface (**j**) weld.

**Figure 12:**
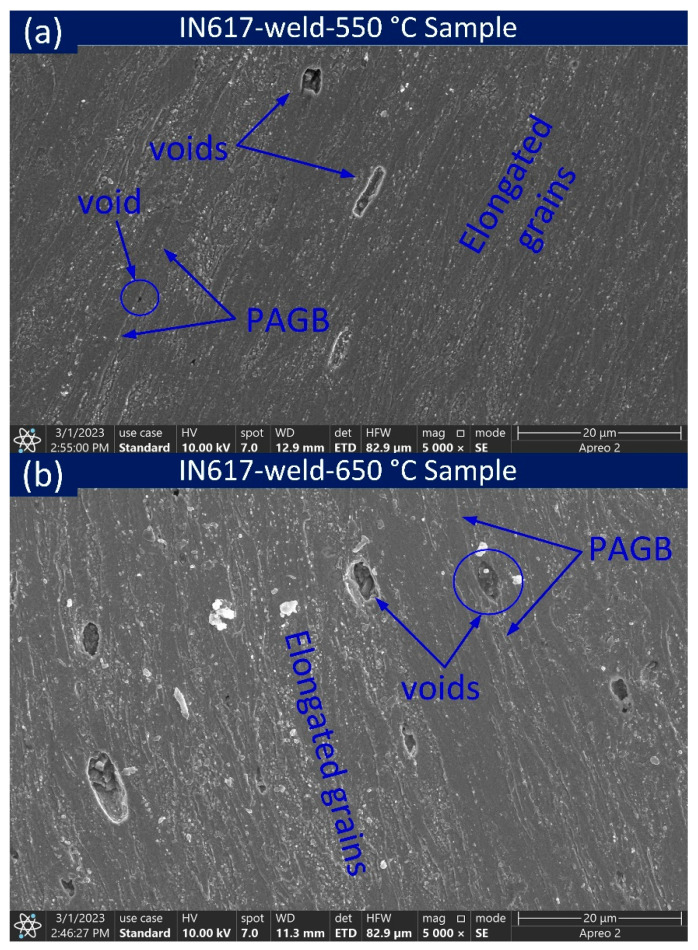
SEM images near fracture location of IN617-weld sample tested at (**a**) 550 °C and (**b**) 650 °C.

An optical light image of the longitudinal section of macroscopic and microscopic characteristics of IN617-weld-550 °C and IN617-weld-650 °C was indicated in Figure 11. The macrograph (Figure 11a,f) indicates the fracture surface was 4.9 mm and 5.2 mm from the respective weld interface of IN617-weld-550 °C and IN617-weld-650 °C, respectively. The large-sized voids were observed just near the fracture location (Figure 11b,g). Near the fracture location, elongated grains and elongated voids were observed and no evidence of appreciable voids or elongation of grain was observed at the HAZ, interface, and weld of IN617-weld-550 °C (Figure 11c–e) and IN617-weld-650 °C (Figure 11h–j). Additionally, the voids and elongated grains seen in the optical image near the fracture are confirmed in the SEM image. Both coarse and fine voids were observed near the fracture locations. In ICHAZ, near the fracture location, fine voids were observed over the PAGBs, confirmed by the SEM images (Figure 12a) for IN617-weld-550 °C, and for IN617-weld-650 °C, comparatively coarse voids were noticed on the PAGBs (Figure 12b). The microstructure in Figure 12 also illustrated large particles distributed near the voids and along the grain boundaries.

### 3.3. Hardness Characteristic

**Figure 13:**
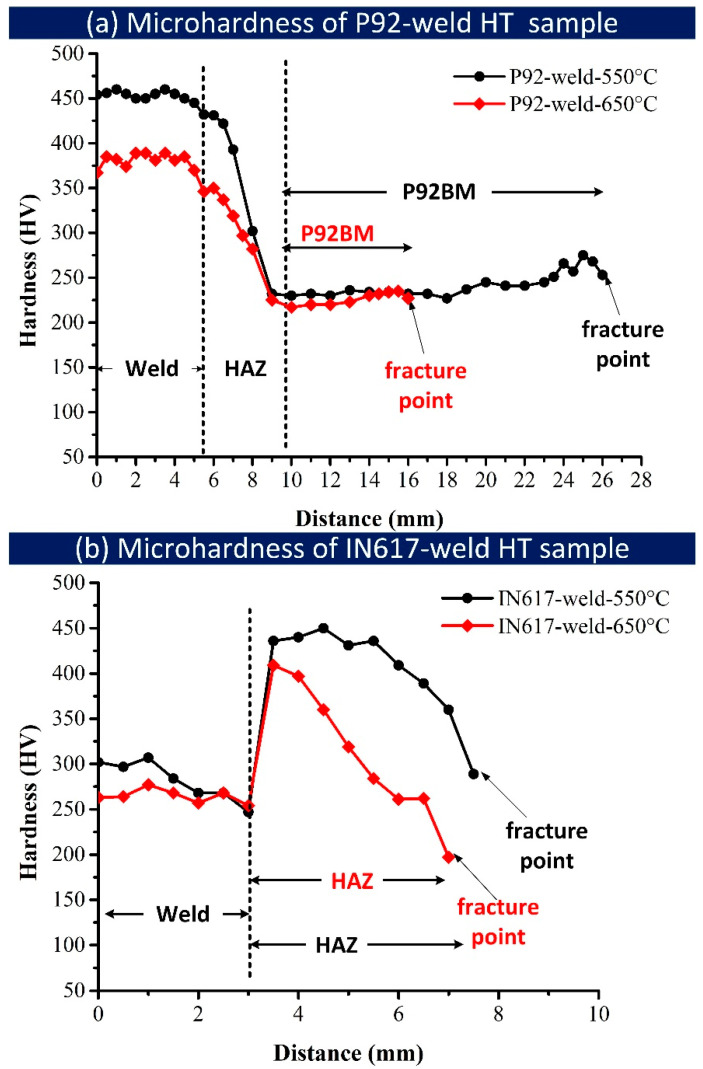
Hardness variation along the longitudinal section from weld centre to near-fracture location HT tensile sample of (**a**) P92-weld and (**b**) IN617-weld.

Based on the as-welded sample hardness profile for both welded plates (P92-weld and IN617-weld), the highest hardness was noticed in the position just near the fusion interface, i.e., CGHAZ, while the lower value of hardness was observed in ICHAZ. It can be seen that ICHAZ was the weakest region of the weldments (Figure 3a). However, the P92-weld sample failed from P92 BM instead of the ICHAZ, whereas in the IN617-weld sample, failure was observed at ICHAZ. The macrograph of the longitudinal section of the tested sample of the P92-weld indicates (Figure 8a,g) that the location of HAZ is very far from the fracture location, whereas the macrograph of the longitudinal section of the tested sample of the IN617-weld indicates (Figure 11a,f) that the fracture location is ICHAZ of P92. The hardness variation along the longitudinal section of the tested samples (P92-weld and IN617-weld) from the weld centre to the near-fracture location is depicted in Figure 13a,b. The sample of P92-weld tested at 550 °C indicates comparatively more hardness values of respective zones (weld, HAZ, 92BM) than the sample tested at 650 °C. The P92-weld displayed the highest hardness in the weld and the lowest at the outer edge of the HAZ and fracture location. The hardness of weld metal in P92-weld-550 °C and P92-weld-650 °C was 454 ± 4 HV and 382 ± 7 HV, respectively. The reduction in hardness of other regions of the weldments was also observed. The reduction in hardness might be due to the tempering reaction, which is a function of the operating temperature and time. For the IN-617 weld, a lower hardness was measured for the weld metal tested at 650 °C as compared to the sample tested at 550 °C, but the difference was low as compared to the P92-weld. This may be due to the difference in microstructure of the weld metal, i.e., martensitic in the P92-weld and austenitic in the IN617-weld. The weld metal exhibited hardness values of 287 ± 17 HV and 266 ± 7 HV for IN617-weld-550 °C and IN617-weld-650 °C, respectively. For the region of P92 HAZs, a similar trend is observed in the IN617-weld as noticed in the P92-weld.

## 4. Conclusions

This study investigated the tensile rupture behaviour of a dissimilar metal weld joint between P92 steel and Alloy 617 at high temperatures. The welds were fabricated using the GTAW process with ER62S-B9 (P92) and ERNiCrCoMo-1 (IN617) fillers. The as-welded joint showed great heterogeneity in microstructure and hardness along the welded joint for both fillers. Due to the formation of the untempered brittle martensitic microstructure in the weld metal of the ER62S-B9 filler, the hardness of the weld metal measured much higher than the ERNiCrCoMo-1 weld. In both fillers, a large variation in the hardness of P92 HAZ was observed, as it was associated with the peak temperature attained by the HAZ. The sample tested at 550 °C and 650 °C shows the failure either from the P92 BM or soft ICHAZ. In the ERNiCrCoMo-1 weld, the failure was observed in P92 ICHAZ with a tensile strength of 466 MPa and 357 MPa at 550 °C and 650 °C, respectively. In the ER62S-B9 weld, the failure was observed in P92 BM with a tensile strength of 402 MPa and 269 MPa at 550 °C and 650 °C, respectively. The failure in both the welds from P92BM/ICHAZ suggested their applicability for A-USC application. However, in the ER62S-B9 weld, higher hardness and untempered martensitic microstructure may deteriorate the life of the welded joint. Hence, the ERNiCrCoMo-1 weld can be considered the best filler for DMJ of P92 steel and Alloy 617. The fracture study of the crack tip shows the crack nucleation and voids near the lath blocks or boundaries due to the higher concentration of the carbide particles. The percentage of elongation in the ERNiCrCoMo-1 weld measured lower than the ER62S-B9 weld in both conditions, and that is also supported by the FESEM study of the fracture surface.

## Figures and Tables

**Table 1:** Tensile properties of samples.

	RT [23]	550 °C			650 °C		
	P92 BM	P92BM	IN617-Weld	P92-Weld	P92 BM	IN617-Weld	P92-Weld
**UTS (MPa)**	758 ± 6	476	466	402	319	357	269
**Yield strength (MPa)**	520 ± 8	332	230	220	291	245	205
**Elongation (%)**	33 ± 3	8.2	14.4	16	12.5	8.9	14
**Area reduction (%)**	69	58.8	51.5	80	86.8	69.7	91.2
**Fracture location**	**-**	**-**	**P92 ICHAZ**	**P92 BM**	**-**	**P92 ICHAZ**	**P92 BM**

## Data Availability

Not applicable.
